# The Challenge of Conserving Amphibian Megadiversity in Madagascar

**DOI:** 10.1371/journal.pbio.0060118

**Published:** 2008-05-06

**Authors:** Franco Andreone, Angus I Carpenter, Neil Cox, Louis du Preez, Karen Freeman, Samuel Furrer, Gerardo Garcia, Frank Glaw, Julian Glos, David Knox, Jörn Köhler, Joseph R Mendelson, Vincenzo Mercurio, Russell A Mittermeier, Robin D Moore, Nirhy H. C Rabibisoa, Herilala Randriamahazo, Harison Randrianasolo, Noromalala Rasoamampionona Raminosoa, Olga Ravoahangimalala Ramilijaona, Christopher J Raxworthy, Denis Vallan, Miguel Vences, David R Vieites, Ché Weldon

## Abstract

Highly diverse and so far apparently untouched by emergent diseases, Malagasy frogs nevertheless are threatened by ongoing habitat destruction, making pro-active conservation actions especially important for preserving this unique, pre-decline, amphibian fauna.

Frogs from Madagascar constitute one of the richest groups of amphibian fauna in the world, with currently 238 described species; caecilians and salamanders are absent [1]. Several frog radiations of the island are species-rich and parallel lemurs and tenrecs in their astonishing morphological and ecological diversity. According to the Global Amphibian Assessment (GAA), Madagascar ranks as the country with the 12th highest amphibian species richness [2,3] (see also http://www.globalamphibians.org), but this is likely an underestimate, because an additional 182 candidate species have been identified since [1]. Diversity is concentrated in rainforests and can locally reach over 100 species. Impressively, 100% of the autochthonous species and 88% of the genera are strictly endemic to Madagascar and its inshore islands [1]. Most of these species belong to two radiations of astonishing ecomorphological and reproductive diversity, the mantellids and the scaphiophrynine plus cophyline microhylids [4,5] (Figure 1).

**Figure 1:**
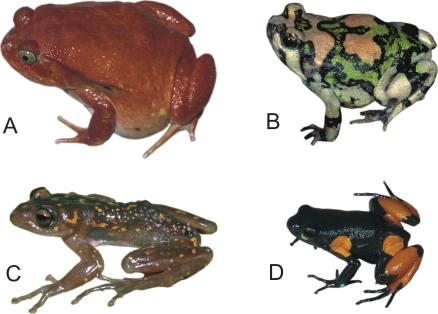
Four Prominent Representatives of Madagascar's Amphibians (A) Dyscophus antongilii (Near Threatened), (B) Scaphiophryne gottlebei (Critically Endangered), (C) Boophis williamsi (Critically Endangered), (D) Mantella cowani (Critically Endangered).

So far, no extinctions of amphibian species have been reported from Madagascar, and chytridiomycosis, a threat for amphibians globally [6,7], has not been detected [8]. Of 220 species assessed by the World Conservation Union (IUCN), nine are listed as Critically Endangered, 21 Endangered, and 25 Vulnerable [3]. This proportion of 25% threatened species is higher than the per-country average of 12%, but lower than that detected globally (32%) and those in various other amphibian hot spots such as Sri Lanka (63%), Mexico (54%), Ecuador (37%), or Colombia (30%) [2].

At first glance, it might seem paradoxical to advocate amphibian conservation actions for a place where catastrophic declines have not yet been detected. However, we argue here that the unique combination of three factors qualifies Madagascar as a top priority for amphibian conservation: (a) an endemic, diverse amphibian fauna, as yet unaffected by emergent diseases, exists; (b) heavy anthropogenic pressures are put on the remaining primary vegetation and amphibian populations; and (c) a strong commitment of the national government to improve conserving biodiversity is present. In other words, Madagascar represents a tractable opportunity to apply what has been learned from the devastated amphibian faunas of areas such as the Neotropics and Australia. In Madagascar, amphibian conservation efforts have the possibility of being pro-active, rather than reactive, or simply postmortem.

Extinctions of Malagasy amphibians have not yet been detected: in fact, all historically described species have been observed during the past 15 years [3], most in the past 5 years. New populations of rare species are discovered at a constant pace, even if some of them are in small forest fragments. Over 500 frog specimens of almost 80 species sampled from most of Madagascar's biogeographic regions and elevational zones tested negative for amphibian chytrid infection using both histological and molecular techniques [8]. So far, no amphibian die-offs have been reported, despite a very high incidence of field work by specialized researchers in recent years. This indicates that the amphibian chytrid fungus is probably absent from Madagascar. The fact that all inventoried forests and forest fragments in Madagascar harbor amphibian faunas rich in species and individuals confirms that amphibian declines due to emergent diseases have probably not recently struck the island.

Nevertheless, the conservation status of Malagasy amphibians is reason for concern. Ongoing habitat destruction has already led to destruction of 90% of the original vegetation and threatens most species [9,10]. Warming trends in Madagascar equal or exceed global averages, and appear to be driving species upslope 19–51 m per decade [11]. This is a particular problem for montane endemics, which are restricted to narrow elevations close to the summits of most of the major massifs in Madagascar [12]. Several species experience high levels of demand in the international pet trade, and over-collecting may represent a confounding threat where habitats are already compromised and populations are small [13]. Accidental human-assisted introduction of amphibian chytrid may have catastrophic effects on the amphibian fauna. Hence, without intensive conservation efforts, Malagasy amphibians may be on the edge of a drastic decline. Here we posit that unprecedented pro-active efforts of habitat protection and vigilance against pathogens may avert an otherwise predictable catastrophic loss of biodiversity.

An important and timely opportunity for conservation in Madagascar exists since President Marc Ravalomanana announced at the Durban World Park Congress in 2003 a commitment to triple the surface of Madagascar's network of protected areas. This declaration gave rise to the so-called Madagascar Action Plan, a political process which is generating strategies for development and conservation. Since 2003, the protected area network has nearly been doubled, and an opportunity exists now to protect small fragments of remaining habitat that are critical for many restricted-range endemic amphibians (Figure 2). A proposal for new candidate areas to protect, based on a high-resolution multi-taxonomic analysis of plants, invertebrates, mammals, reptiles, and amphibians, has been developed [14]. This study suggests that multi-taxonomic rather than single-taxon approaches are critical for identifying areas likely to promote the persistence of most species; hence, the areas identified in this study will benefit not only amphibians but other groups as well.

**Figure 2:**
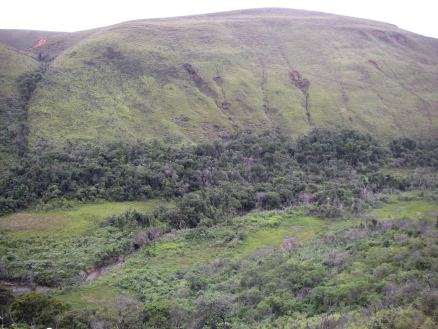
Habitat Fragments as Last Refuges for Amphibian Diversity In many largely deforested areas of Madagascar, small habitat fragments remain, which harbor significant amphibian diversity, and may allow the survival of yet undiscovered range-restricted species. So far no amphibian extinctions have been detected for Madagascar. A survey of the pictured forest fragment west of Ambatondrazaka in 2008 yielded 12 frog species, one of which is new to science.

This process also contributed to trigger the workshop *A Conservation Strategy for the Amphibians of Madagascar* (ACSAM), held in Antananarivo in September, 2006, which was attended by more than 100 biologists and major conservation and governmental agencies [15]. Participants agreed on the urgency of defining priority areas for amphibian conservation. Currently, the habitat of various Critically Endangered amphibian species (e.g., Mantella cowani, Boophis williamsi and Mantidactylus pauliani) is just located at high-altitude sites and not included in any existing reserve nor in the new areas already earmarked for protected area creation by the “Durban Vision” process. ACSAM thus represents an important step in national implementation of the Amphibian Conservation Action Plan (ACAP) [16,17].

The initial results of this workshop are now emerging. To provide a scientific basis for conservation actions, a Proceedings volume will be published in early 2008, summarizing the conservation-relevant scientific results of the workshop in 26 articles by 69 authors [18]. To increase local capacity and awareness, Conservation International produced the first of a pocket field guide series on priority species [19], and more are planned. The Amphibian Specialist Group (ASG) has published a popular multilingual booklet on Madagascar amphibian conservation [20]. And an updated edition of a field guide has been translated into Malagasy [1,21]. With accounts on all species of Malagasy amphibians on 528 pages, this is the first comprehensive local-language book on Madagascar's biodiversity.

A further crucial step has been the designation of a Malagasy Amphibian Executive Secretary, based in Antananarivo, who is now working with the ASG to coordinate amphibian conservation activities. The ASG will work with the national government to implement quarantine measures related to commercial trade in aquarium fishes and plants to prevent the accidental introduction of amphibian chytrid. To ensure a rapid detection of possible declines or die-offs, amphibian monitoring in the Menabe region and in the Ranomafana National Park will be coordinated by the Durrell Wildlife Conservation Trust and by the Tropical Ecology Assessment and Monitoring Network (http:// www.teaminitiative.org). A variety of amphibian species from Madagascar will be exposed in a South African lab to chytrid infection, to understand their susceptibility to chytridiomycosis. In collaboration with the Amphibian Ark initiative, an agreement has been reached for promoting tailored captive breeding programs, and target species have been prioritized (http://zims.isis.org/aark/). The experiences acquired in these initial captive-breeding efforts will be crucial if amphibian chytrid reaches the island despite efforts to prevent its introduction. An immediate implementation of large-scale, captive-breeding efforts as an extension of the Amphibian Ark [7] (http://www.amphibianark.org) will become necessary in such a case to ensure survival of the bulk of the Malagasy amphibian species until protocols to induce resistance or inoculate frogs with bacterial antifungal peptides can be applied [7,22,23].

An amphibian action plan for Madagascar has now been developed that includes precise yearly budgets for future actions [24]. Unconventional funding sources are being explored, such as name auctions for undescribed species (http://www.biopat.de), which currently support a conservation program for the tomato frog, Dyscophus antongilii. However, significant investments from major conservation agencies will also be necessary to generate momentum. Madagascar may be the only worldwide amphibian diversity hot spot still in a pre-decline phase where intensive pro-active conservation measures are feasible, and where the impacts of climate change can be measured without the confounding influences of emergent diseases such as chytrid fungus [11,25]. This opportunity to preserve a globally significant and intact amphibian sanctuary should not be missed, yet ironically, its pre-decline status could actually hinder timely conservation action being taken. We thus advocate urgency rather than complacency towards implementing a comprehensive conservation initiative for the Malagasy amphibian fauna.

## Abbreviations

ASG: Amphibian Speciality Group
